# S100B Impairs Oligodendrogenesis and Myelin Repair Following Demyelination Through RAGE Engagement

**DOI:** 10.3389/fncel.2020.00279

**Published:** 2020-09-04

**Authors:** Gisela Santos, Andreia Barateiro, Dora Brites, Adelaide Fernandes

**Affiliations:** ^1^Neuron Glia Biology in Health and Disease, Research Institute for Medicines (iMed.ULisboa), Faculty of Pharmacy, Universidade de Lisboa, Lisboa, Portugal; ^2^Department of Biochemistry and Human Biology, Faculty of Pharmacy, Universidade de Lisboa, Lisboa, Portugal

**Keywords:** demyelination, inflammation, neurodegeneration, oligodendrogenesis, receptor for advanced glycation end products (RAGE), remyelination, S100B

## Abstract

Increased expression of S100B and its specific receptor for advanced glycation end products (RAGE) has been described in patients with multiple sclerosis (MS), being associated with an active demyelinating process. We previously showed that a direct neutralization of S100B reduces lysophosphatidylcholine (LPC)-induced demyelination and inflammation using an *ex vivo* demyelinating model. However, whether S100B actions occur through RAGE and how oligodendrogenesis and remyelination are affected are not clarified. To evaluate the role of the S100B–RAGE axis in the course of a demyelinating insult, organotypic cerebellar slice cultures (OCSC) were demyelinated with LPC in the presence or absence of RAGE antagonist FPS-ZM1. Then, we explored the effects of the S100B–RAGE axis inhibition on glia reactivity and inflammation, myelination and neuronal integrity, and on oligodendrogenesis and remyelination. In the present study, we confirmed that LPC-induced demyelination increased S100B and RAGE expression, while RAGE antagonist FPS-ZM1 markedly reduced their content and altered RAGE cellular localization. Furthermore, FPS-ZM1 prevented LPC-induced microgliosis and astrogliosis, as well as NF-κB activation and pro-inflammatory cytokine gene expression. In addition, RAGE antagonist reduced LPC-induced demyelination having a beneficial effect on axonal and synaptic protein preservation. We have also observed that RAGE engagement is needed for LPC-induced oligodendrocyte (OL) maturation arrest and loss of mature myelinating OL, with these phenomena being prevented by FPS-ZM1. Our data suggest that increased levels of mature OL in the presence of FPS-ZM1 are related to increased expression of microRNAs (miRs) associated with OL differentiation and remyelination, such as miR-23a, miR-219a, and miR-338, which are defective upon LPC incubation. Finally, our electron microscopy data show that inhibition of the S100B–RAGE axis prevents axonal damage and myelin loss, in parallel with enhanced functional remyelination, as observed by the presence of thinner myelin sheaths when compared with Control. Overall, our data implicate the S100B–RAGE axis in the extent of myelin and neuronal damage, as well as in the inflammatory response that follows a demyelinating insult. Thus, prevention of RAGE engagement may represent a novel strategy for promoting not only inflammatory reduction but also neuronal and myelin preservation and/or remyelination, improving recovery in a demyelinating condition as MS.

## Introduction

Multiple sclerosis (MS) is the most common chronic inflammatory demyelinating disease of the central nervous system (CNS), characterized by focal areas of inflammation and demyelinated areas, extensive axonal injury (Bjartmar et al., 2001), neuronal loss (Filippi and Rocca, 2005), and synaptic alterations (Centonze et al., 2009). In MS early inflammatory stages, activated immune cells highly produce pro-inflammatory factors including interleukin-1β (IL-1β) and tumor necrosis factor-α (TNF-α), which not only are cytotoxic but also induce glial reactivity leading to an exacerbated local inflammation, including the expression of astroglial S100B protein.

S100B, a small Ca^2+^-binding protein, highly expressed in the CNS following injury, exerts both intra- and extracellular roles. Within cells, S100B is involved in the regulation of both proliferation and differentiation of neurons and glia (Donato et al., 2009; Sorci et al., 2010a). Extracellularly, S100B has a dual function in a concentration-dependent manner (Van Eldik and Wainwright, 2003), with additional evidences suggesting also a multimeric-dependent S100B binding to the receptor for advanced glycation end products (RAGE; Ostendorp et al., 2007). At physiological levels, in the nanomolar range, S100B promotes cell proliferation and migration but inhibits apoptosis and differentiation (Sorci et al., 2010b). Indeed, such S100B levels modulate neurite outgrowth, synaptogenesis, and long-term plasticity (Arcuri et al., 2005), while preventing astrocytic (Brozzi et al., 2009) and microglial activation and consequent inflammation (Zhang et al., 2011). Notwithstanding, elevated S100B concentrations, in the micromolar range, exacerbate the inflammatory response through glial activation and subsequent release of inflammatory cytokines and stress-related enzymes, culminating in cell dysfunction and death (Bianchi et al., 2010; Sorci et al., 2010a).

Concerning oligodendrogenesis, S100B is expressed by oligodendrocyte precursor cells (OPC) and needed for their differentiation and maturation in myelinating oligodendrocytes (OL). In fact, S100B absence impairs the morphological maturation of pre-OL cells *in vitro*, whereas S100B^−/−^ mice showed a delayed OPC maturation following a demyelinating insult (Deloulme et al., 2004). Besides, S100B is associated with microtubular structures in cultured OL (LoPresti, 2002), being implicated in their cytoskeleton dynamics (Richter-Landsberg and Heinrich, 1995), which is critical for correct myelination. However, excessive S100B levels arrest oligodendrogenesis and OL maturation *in vitro* and *de novo* myelination in an *ex vivo* model (Santos et al., 2018). Accordingly, increased S100B levels in the brain have been associated with numerous disorders with altered myelination. We reported the presence of excessive S100B in the cerebrospinal fluid (CSF) and serum of MS patients at diagnosis, as well as in active lesions of *post-mortem* brain samples (Barateiro et al., 2016). Additionally, S100B levels have been suggested to stratify the different stages of the disease, with the highest concentrations being found in relapsing remitting MS patients in opposite to lowest levels in progressive MS (Bartosik-Psujek et al., 2011; Barateiro et al., 2016). S100B has also been indicated to monitor treatment efficacy, since S100B concentration significantly decreases after therapy (O’Connell et al., 2014). Moreover, using an *ex vivo* model, we observed a high expression and secretion of S100B upon demyelination and that S100B blockade with a specific antibody reduced demyelination, astrocyte reactivity, and gene expression of pro-inflammatory cytokines (Barateiro et al., 2016). Interestingly, active MS lesions also showed an increase of RAGE expression by microglia/macrophage cells (Barateiro et al., 2016), suggesting that S100B may be exerting its detrimental effects on MS pathogenesis through RAGE engagement.

In the present study, we examined how the use of a RAGE antagonist, the FPS-ZM1, which inhibits RAGE engagement by S100B, could prevent the damage observed in our *ex vivo* demyelinating model. Our results clearly demonstrate that inhibition of S100B–RAGE interaction may have a dual role in both the protection of myelin sheath upon lysophosphatidylcholine (LPC)-induced demyelination, as well as in the resolution of the inflammatory milieu favoring a faster remyelination and preservation of neuronal structures, further supporting a critical role of this axis in MS pathogenesis.

## Materials and Methods

### Animals

Animal care followed the recommendations of the European Convention for the Protection of Vertebrate Animals Used for Experimental and Other Scientific Purposes (Council Directive 86/609/EEC) and National Law 1005/92 (rules for protection of experimental animals). All animal procedures were approved by the Institutional animal care and use committee. Every effort was made to minimize the number of animals used and their suffering.

### *Ex vivo* Model of Demyelination

To study the role of the S100B–RAGE axis in a demyelinating event, we used organotypic cerebellar slice cultures (OCSC) treated with LPC as usual in our laboratory, which was shown to induce a great release of S100B (Barateiro et al., 2016). Briefly, cerebellum of rat pups at postnatal day 10 were isolated and cut in parasagittal slices of 400 μm using a McIlwain tissue chopper. Four slices of different animals were placed into a 0.4-μm pore membrane culture insert (BD Falcon, Lincoln Park, NJ, USA) in six-well cell culture plates, in an air–liquid interface, at 37°C and 5% CO_2_ conditioned atmosphere and kept in culture until 7 days *in vitro* (DIV) to allow the clearance of debris and full myelination (Birgbauer et al., 2004). For the first 3 DIV, slices were cultured with 1 ml per well of culture medium consisting of 50% minimal essential media (MEM; Gibco, Life Technologies, Inc., Grand Islands, NY, USA), 25% of both heat-inactivated horse serum (Gibco) and Earle’s balanced salt solution (EBSS, Gibco), 6.5 mg/ml glucose, 36 mM HEPES (Biochrom AG, Berlin, Germany), and 1% of both L-glutamine (Sigma–Aldrich, St. Louis, MO, USA) and 1% of antibiotic–antimycotic (Sigma–Aldrich). Half of the culture medium was renewed every day. At 4 DIV, to improve neuronal viability, culture medium was totally replaced by 1 ml of a serum-free medium, containing Neurobasal-A (NB, Gibco), supplemented with 2% B-27 (Gibco), 1% L-glutamine, 36 mM glucose, 1% of antibiotic–antimycotic, and 25 mM HEPES. Again, half of the medium was daily replaced by new medium.

After 7 DIV, slices were incubated with 0.5 mg/ml LPC in serum-free culture medium during 18 h, at 37°C, in the presence or absence of a RAGE antagonist FPS-ZM1 (Calbiochem, San Diego, CA, USA) with *K*_i_ = 230 nM for S100B–RAGE interaction inhibition (Deane et al., 2012). We used FPS-ZM1 in a concentration of 3 μM, more than 10× the *K*_i_, to assure a maximal inhibition of S100B interaction with RAGE under non-toxic conditions. Following incubation, slices were maintained in fresh medium or in medium supplemented with FPS-ZM1 during 30 h. Slices were collected at 9 DIV and stored in RIPA (radio-immunoprecipitation assay buffer) for protein extraction, TRIzol^®^ reagent (Invitrogen, Carlsbad, CA, USA) at −20°C for RNA extraction, fixed in 4% paraformaldehyde for immunohistochemistry assays, or fixed in 4% paraformaldehyde and 2% glutaraldehyde in 0.1 M phosphate buffer (PB) for electron microscopy.

### Immunocytochemistry and Data Analysis

Fixed cerebellar slices were incubated with blocking solution (1 nM HEPES, 2% heat-inactivated horse serum, 10% heat-inactivated fetal bovine serum, 1% bovine serum albumin, and 0.25% Triton X-100 in Hank’s Balanced Salt Solution) for 3 h at room temperature. Slices were then incubated with primary antibodies diluted in blocking solution for 24 h at 4°C. The following antibodies were used: neurofilament medium (NF-160, mouse, 1:200, Novocastra, Wetzlar, Germany) for neuronal axons, neural-glial antigen 2 (NG2, rabbit, 1:200, Merck Millipore, Billerica, MA, USA) for OPC, myelin basic protein (MBP, rat, 1:200, Serotec, Raleigh, NC, USA) for mature OL, glial fibrillary acidic protein (GFAP, mouse, 1:200, Novocastra) for astrocytes, anti-ionized calcium binding adaptor molecule 1 (Iba1, rabbit, 1:250, Wako, Richmond, VA, USA) and arginase-1 (Arg-1, goat, 1:50, Santa Cruz Biotechnology, Santa Cruz, CA, USA) for microglia, S100B (rabbit, 1:200, Abcam, Cambridge, UK), and RAGE (rabbit, 1:100, Abcam). After, slices were incubated with secondary antibodies: Alexa 594 anti-rat, Alexa 488 anti-mouse, Alexa 488 anti-rabbit, or Alexa 594 anti-goat (1:1,000, Invitrogen), in blocking solution for 24 h at 4°C. To identify the total number of cells, nuclei were stained with Hoechst 33258 dye (1:1,000, Sigma–Aldrich). Fluorescent images of the myelin tracts in the white matter were acquired using a Confocal Point Scanning Microscope Zeiss LSM 710 META (Zeiss, Germany). The range of the slice was determined using Z-stack imaging at 1-μm intervals, and a series of images derived from merged Z-stack imaging were analyzed. The percentage of fluorescent occupancy for each antibody was measured. A cut off intensity threshold was defined for each region of interest (ROI), which corresponds to a minimum intensity due to specific staining above background values. Then, the percentage of fluorescent occupancy of GFAP, Iba1, NG2, MBP, and NF-160 above such threshold was measured automatically using ImageJ software in each cerebellum region and normalized to the Control. To determine S100B to GFAP and RAGE to Arg-1 colocalization, a cut off intensity threshold for each marker was defined as ROI and colocalization of two ROI (S100B+GFAP or RAGE+Arg-1) measured in ImageJ. In addition, RAGE expression was assessed at nuclei and cytoplasm by surrounding either the nuclei in Hoechst or total cell in Arg-1 channel and measuring RAGE expression at RAGE channel, and defining the cytoplasmic intensity as total cell minus nuclear staining. Regarding myelination, the index of myelinated fibers was obtained by the ratio between the area of colocalization of NF-160 with MBP and the total area occupied by NF-160. Results are given by mean values from 10 separate microscopic fields of slices from at least four different animals.

### Gene and microRNA Expression

Total RNA was isolated from treated slices using the TRIzol^®^ reagent method according to the manufacturer’s instructions and RNA concentration was quantified using Nanodrop ND-100 Spectrophotometer (NanoDrop Technologies, Wilmington, DE, USA). For gene expression, aliquots of 300 ng of total RNA were reversely transcribed using the SensiFAST^TM^ cDNA Synthesis Kit (Bioline, MA, USA), under recommended conditions. qRealTime-PCR was performed on a real-time PCR detection system (Applied Biosystems 7300 Fast Real-time PCR System, Applied Biosystem, Madrid, Spain) using a SensiFAST^TM^ SYBR No-ROX Kit (Bioline), as usual in our laboratory (Santos et al., 2018). Primer sequences used for qRealTime-PCR analysis are listed in Table 1. The reaction conditions consisted of polymerase activation/denaturation and well-factor determination at 95°C for 10 min, followed by 50 amplification cycles at 95°C for 10 s and 60°C for 1 min (ramp rate 1.6°C/s). Relative mRNA concentrations were calculated using the ΔΔCT equation, and quantification of genes was made in comparison to an endogenous control, β-actin, for each experiment.

**Table 1:** List of pairs of primers used to determine gene expression by qRealTime-PCR assays.

Gene	Forward	Reverse
HMGB1	ctcagagaggtggaagaccatgt	gggatgtaggttttcatttctctttc
IL-1β	caggctccgagatgaacaac	ggtggagagctttcagctcata
MBP	agtcgcagaggacccaagat	gacaggcctctcccctttc
NG2	gggctgtgctgtctgttga	tgattcccttcaggtaaggca
PDGFRα	acgttcaagaccagcgagtt	cagtttgatggacgggagtt
PLP	cacttacagcaggtgattagagg	aaacaagagataaacaactggga
PSD-95	cgaggatgccgtggcagcc	catggctgtggggtagtcagtgcc
RAGE	tgggcaccatcttcatcattc	ggtcacccagcacaccactt
S100B	acccacatctggcagaatgag	agccatgacctttcgcattag
Synaptophysin	tcaggactcaacacctcagtgg	aacacgaaccataagttgccaa
TNF-α	tactgaacttcggggtgattggtcc	cagccttgtcccttgaagagaacc
β-actin	gctccggcatgtgcaa	aggatcttcatgaggtagt

*All primers were purchased from Thermo Fisher Scientific, Waltham, MA, USA. HMGB1, high mobility group box 1; IL-1β, interleukin-1β; MBP, myelin basic protein; NG2, neural–glial antigen 2; PDGFRα, platelet-derived growth factor receptor α; PLP, myelin proteolipid protein; PSD-95, postsynaptic density protein-95; RAGE, receptor for advanced glycation end products; TNF-α, tumor necrosis factor-α*.

For microRNA (miR) analysis, conversion of cDNA was achieved from 20 ng of total RNA with the universal cDNA Synthesis Kit (Qiagen, Hilden, Germany) as usual (Caldeira et al., 2017), following manufacturer’s recommendations. The miRCURY LNA Universal RT miR PCR system (Qiagen) was used in combination with predesigned primers (Qiagen) for specific target sequences listed in Table 2. The reaction conditions consisted of polymerase activation/denaturation and well-factor determination at 95°C for 10 min, followed by 50 amplification cycles at 95°C for 10 s and 60°C for 1 min (ramp rate 1.6°C/s). Relative mRNA concentrations were calculated using the ΔΔCT equation, in comparison to the geometric average of the two reference genes, SNORD110 and RNU1A1. UniSp6–RNA spike-in Control was used to monitor PCR efficiency. Two readings were performed for each sample.

**Table 2:** List of target sequences used to determine microRNA expression in qRealTime-PCR.

miRs	Target sequence (5′–3′)
hsa-miR-23a-3p	AUCACAUUGCCAGGGAUUUCC
hsa-miR-219a-5p	UGAUUGUCCAAACGCAAUUCU
rno-miR-338-3p	UCCAGCAUCAGUGAUUUUGUUGA

*All primers were purchased from Qiagen, Hilden, Germany. hsa—Human (*Homo sapiens*) origin but also validated for rat specimens, rno—*Rattus norvegicus*; SNORD110 and RNU1A1 were used as a reference gene (human, mouse, and rat); UniSp6—RNA spike-in Control was used to monitor PCR efficiency. miR, microRNA*.

### Western Blot Analysis

Total protein extracts from slices were obtained by lysing slices in RIPA buffer, followed by sonication and centrifugation at 12,000 *g* for 10 min. Total protein concentrations were measured using Nanodrop ND-100 Spectrophotometer, and Western blot was carried out as usual in our lab (Santos et al., 2018). The primary antibodies were as follows: NG2 (rabbit, 1:250, Merck Millipore), MBP (rat, 1:250, Serotec), S100B (rabbit, 1:500, Abcam), RAGE (rabbit, 1:800, Abcam), GFAP (rabbit, 1:500, NovoCastra), high-mobility group box 1 (HMGB1, mouse, 1:200, BioLegend), phosphorilated nuclear factor-κB (pNF-κB rabbit, 1:500, Abcam), NF-κB (rabbit, 1:500, Santa Cruz Biotechnology), or β-actin (mouse, 1:10,000; Sigma). Protein bands were detected using WesternBright Sirius reagent (Advansta, Menlo Park, CA, USA) and visualized using ChemiDoc^TM^ XRS System (Bio-Rad, Hercules, CA, USA). Results were normalized to β-actin expression for each experiment.

### Resin Embedding, Semi-thin Sections, and Electron Microscopy

Electron microscopy was used to confirm the integrity of myelin and to assess actual remyelination as documented by thin myelin sheaths. Cerebellar slices were fixed overnight at 4°C using a solution of 4% paraformaldehyde and 2% glutaraldehyde in 0.1 M PB and then washed three times with 0.1 M PB. Slices were cut off into smaller pieces in order to have only regions associated with the myelin tracts and for chemical infiltration purposes. Samples were subsequently post-fixed with 1% osmium tetroxide in 0.1 M PB reduced with 0.8% potassium ferrocyanide for 45 min and stained with 1% tannic acid for 20 min; both steps were performed at 4°C. En bloc staining was done using 0.5% uranyl acetate for 1 h at room temperature. Samples were dehydrated with successive 10-min washes in increasing concentrations of ethanol. Slices were infiltrated for 1 h with 1:3, 1:2, and 1:1 Epon:ethanol mixture, followed by infiltration with pure Epon for 2 h. Slices were embedded in Epon in a plastic BEEM capsule. The sections of 70 nm were cut onto slot copper/palladium grids coated with 1% formvar in chloroform and stained with 2% uranyl acetate and Reynold’s lead citrate, for 5 min each. Grids were imaged using a Hitachi H-7650 operating at 100 keV equipped with an XR41M mid mount AMT digital camera. Images were taken only from regions associated with myelin tracts where it is possible to clearly observe axonal bundles. Percentage of myelinated axons, axon diameter, myelin thickness, and standard *g*-ratio were calculated from measured area based on assumption of circularity using Fiji, with a minimum of 200 axons per sample analyzed. *g*-ratio was calculated by the ratio between the diameter to the innermost compact myelin layer and the diameter to the outermost compact myelin layer. Myelin thickness was calculated by subtracting the axonal diameter from the diameter of the outermost compact layer.

### S100B Determination

Determination of S100B concentration was performed by in-house enzyme-linked immunosorbent assay (ELISA) as usual in our laboratory (Barateiro et al., 2016). Briefly, incubation media samples were incubated overnight at 4°C on a 96-well plate previously coated with a monoclonal anti-S100B antibody (2.5 μg/ml, Abnova, Taiwan). Thereafter, biotinylated mouse monoclonal anti-S100B antibody (2.5 μg/ml, Abnova) was added and incubated for 2 h at room temperature. Finally, Streptavidin–HRP (1:5,000, Sigma–Aldrich) was added for another 1 h at room temperature. The colorimetric reaction with TMB^®^ (Sigma–Aldrich) was measured at 450 nm in a microplate absorbance spectrophotometer.

### Statistical Analysis

All results are presented as mean ± SEM. Differences between groups were determined by one-way ANOVA for multiple comparisons and correlations assessed by Pearson correlation coefficient, using GraphPad PRISM 6.0 (GraphPad Software, San Diego, CA, USA), as appropriate. Statistical significance was considered for *p*-value < 0.05.

## Results

### Increased Expression of Both S100B and RAGE by LPC-Induced Demyelination Is Prevented by RAGE Antagonist

**Figure 1:**
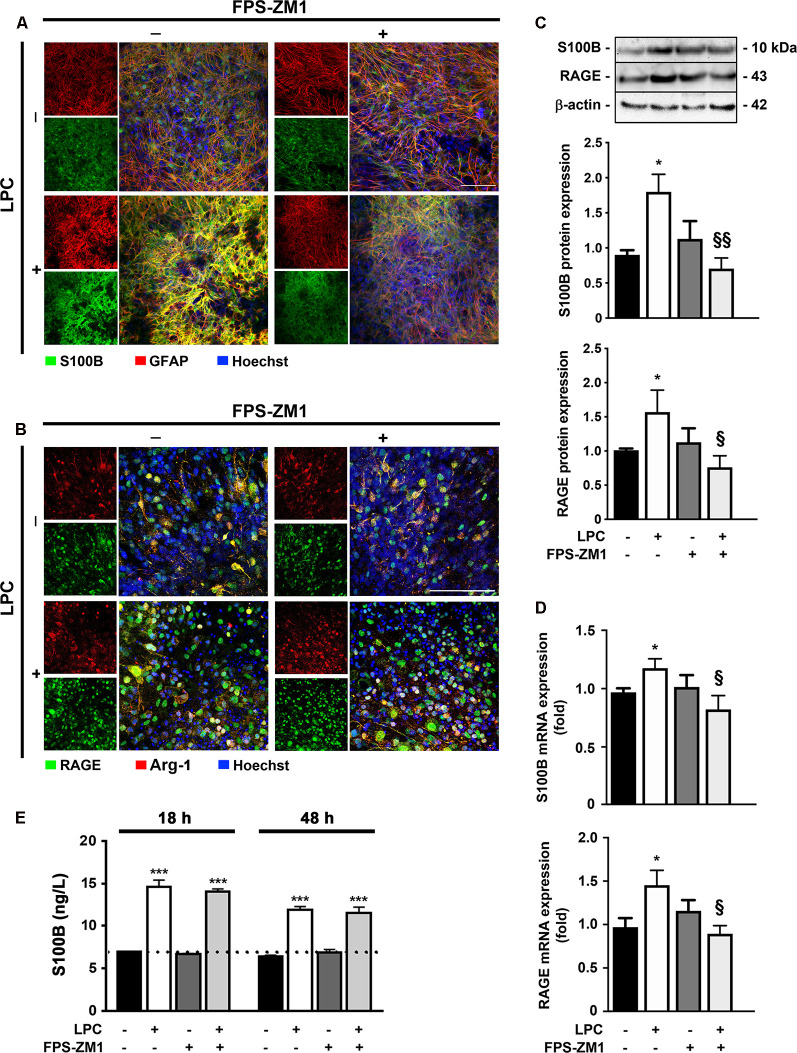
Lysophosphatidylcholine (LPC)-induced demyelination promotes S100B and receptor for advanced glycation end products (RAGE) expression that is reduced by RAGE antagonist. Organotypic cerebellar slice cultures (OCSC) were exposed to LPC (0.5 mg/ml) or LPC plus RAGE antagonist FPS-ZM1 (3 μM) at 7 days *in vitro* for 18 h. Following 30 h of recovery, OCSC were immunostained for **(A)** S100B (green) and astrocytes (glial fibrillary acidic protein, GFAP, red) or for **(B)** RAGE (green) and microglia (arginase-1, Arg-1, red) and stained with Hoechst to detect nuclei (blue). Representative images are shown. Scale bar represents 100 μm. Moreover, expression of S100B and RAGE was also evaluated by **(C)** Western Blot and **(D)** qRealTime-PCR, and secretion of S100B to the incubation media was assessed by enzyme-linked immunosorbent assay (ELISA; **E**). Results are mean ± SEM from at least four independent experiments. One-way ANOVA with Bonferroni multiple comparison test was used for statistical significance (***p < 0.001 and *p < 0.05 vs. Control; ^§§^*p* < 0.01 and ^§^*p* < 0.05 vs. LPC).

It has been described a high release of S100B in demyelinating disorders, and we have previously shown that in MS *post-mortem* active lesions, there is an elevated expression of S100B by astrocytes and of its receptor RAGE by macrophages/microglial cells (Barateiro et al., 2016). So, we first characterized S100B and RAGE expression upon LPC-induced demyelination of OCSC. As shown in Figure 1, we observed that S100B is highly expressed in response to LPC, mainly by astrocytes, as indicated by its colocalization with GFAP (>80%, Supplementary Figure S1A), but reduced upon co-incubation with RAGE antagonist FPS-ZM1 (Figure 1A). RAGE expression was also markedly enhanced upon demyelination (Figure 1B), being mainly localized to microglia, as indicated by its colocalization with Arg-1 (>85%, Supplementary Figure S1B), a typical microglial marker, which, despite being indicated as an M2 microglia phenotype marker in the present model, is able to stain all Iba1+ microglia. Furthermore, RAGE expression, which appeared to be essentially nuclear in Control, shifted to a cytoplasmic and cell membrane localization as indicated by a decrease of nuclear-to-cytoplasm expression ratio (0.4-fold, *p* < 0.05 vs. Control, Supplementary Figures S1C,D). When LPC was co-incubated with FPS-ZM1, RAGE expression was reduced but maintained its cytoplasmic and cell membrane localization. In accordance, protein and gene expression of both markers showed the same pattern (Figures 1C,D). Both protein expression and gene expression of S100B and RAGE were increased 30 h after LPC-induced demyelination (protein: 1.9- and 1.6-fold, *p* < 0.05, respectively; gene: 1.2- and 1.5-fold, *p* < 0.05, respectively), whereas FPS-ZM1 co-incubation abrogated this effect (at least *p* < 0.05). These results indicate that LPC-induced demyelination enhances the expression of both S100B and RAGE, whose axis activation may contribute to associated damage. To further evaluate whether secreted S100B may elicit a feedforward reaction leading to more S100B release *via* RAGE engagement, we next assessed S100B levels in incubation media by ELISA (Figure 1E). As expected, we observed that after LPC incubation (18 h), there was a marked secretion of S100B to the incubation media (2.1-fold, *p* < 0.01) that was maintained after 30 h of recovery (48 h, 2.0-fold, *p* < 0.01). Interestingly, following co-treatment with RAGE antagonist, secreted S100B levels were only slightly lower than those of LPC incubation. Overall, these results suggest that LPC induces a marked S100B secretion, which is able to promote its own mRNA and protein expression still at 48 h, as observed under LPC conditions. However, in the presence of RAGE antagonist, despite the presence of S100B in the media, S100B–RAGE blockade prevents this further S100B expression, potentially preventing additional detrimental effects.

Additionally, other inflammatory molecules, such as HMGB1, also interact with RAGE and can compete with S100B for the receptor binding (Sims et al., 2010). In that sense, we next evaluated HMGB1 protein and gene expression but found no alterations in response to LPC treatment (Supplementary Figure S2), suggesting that in the present model, RAGE engagement is mainly due to S100B binding.

### Gliosis and Inflammatory Response Following LPC-Induced Demyelination Are Prevented by RAGE Antagonist

**Figure 2:**
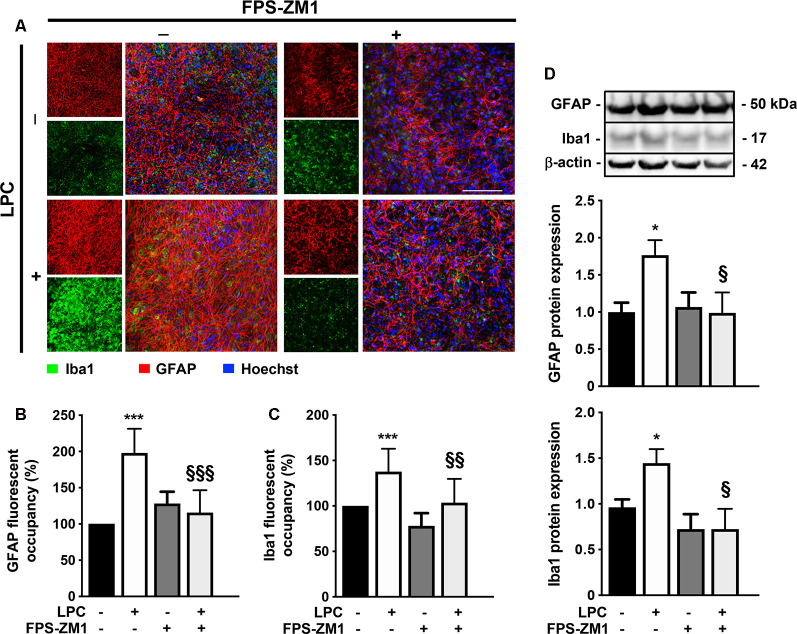
LPC-induced gliosis is prevented by RAGE antagonist. OCSC were exposed to LPC (0.5 mg/ml) or LPC plus RAGE antagonist FPS-ZM1 (3 μM) at 7 days *in vitro* for 18 h. Following 30 h of recovery, OCSC were immunostained for astrocytes (GFAP, red), microglia (Iba1, green) and stained with Hoechst to detect nuclei (blue). **(A)** Representative images are shown. Scale bar represents 100 μm. Gliosis were quantified by measuring the fluorescent occupancy of **(B)** GFAP (astrogliosis) and **(C)** Iba1 (microgliosis). **(D)** Protein expression of GFAP and Iba1 was evaluated by Western blot. Results are mean of fold change vs. Control (± SEM) from at least four independent experiments. One-way ANOVA with Bonferroni multiple comparison test was used for statistical significance (***p < 0.001 and *p < 0.05 vs. Control; ^§§§^*p* < 0.001, ^§§^*p* < 0.01 and ^§^*p* < 0.05 vs. LPC).

Activation and proliferation of microglia and astrocytes and consequent inflammation are observed within MS lesions, suggesting that innate immune response by resident CNS cells might play a critical role in both OL injury and axonal degeneration (Mayo et al., 2012). In this context, we further decided to explore the role of RAGE activation on microgliosis and astrogliosis. As expected, LPC-induced demyelination promoted astrogliosis, as observed by an increase in fluorescent occupancy of GFAP^+^ hypertrophic astrocytes (2.0-fold, *p* < 0.001, Figures 2A,B), which was corroborated by enhanced protein expression (1.8-fold, *p* < 0.05, Figure 2D), both prevented by co-exposure to RAGE antagonist FPS-ZM1 (1.2- and 1.0-fold, *p* < 0.001 and *p* < 0.05 vs. LPC, respectively). Interestingly, LPC also promoted a marked microgliosis, with Iba1^+^ cells showing a more amoeboid morphology that resulted in increased fluorescent occupancy of these cells (1.4-fold, *p* < 0.001, Figures 2A,C) and protein expression (1.4-fold, *p* < 0.05, Figure 2D), which were again blocked by co-treatment with RAGE antagonist (1.0- and 0.7-fold, *p* < 0.01 and *p* < 0.05 vs. LPC, respectively).

**Figure 3:**
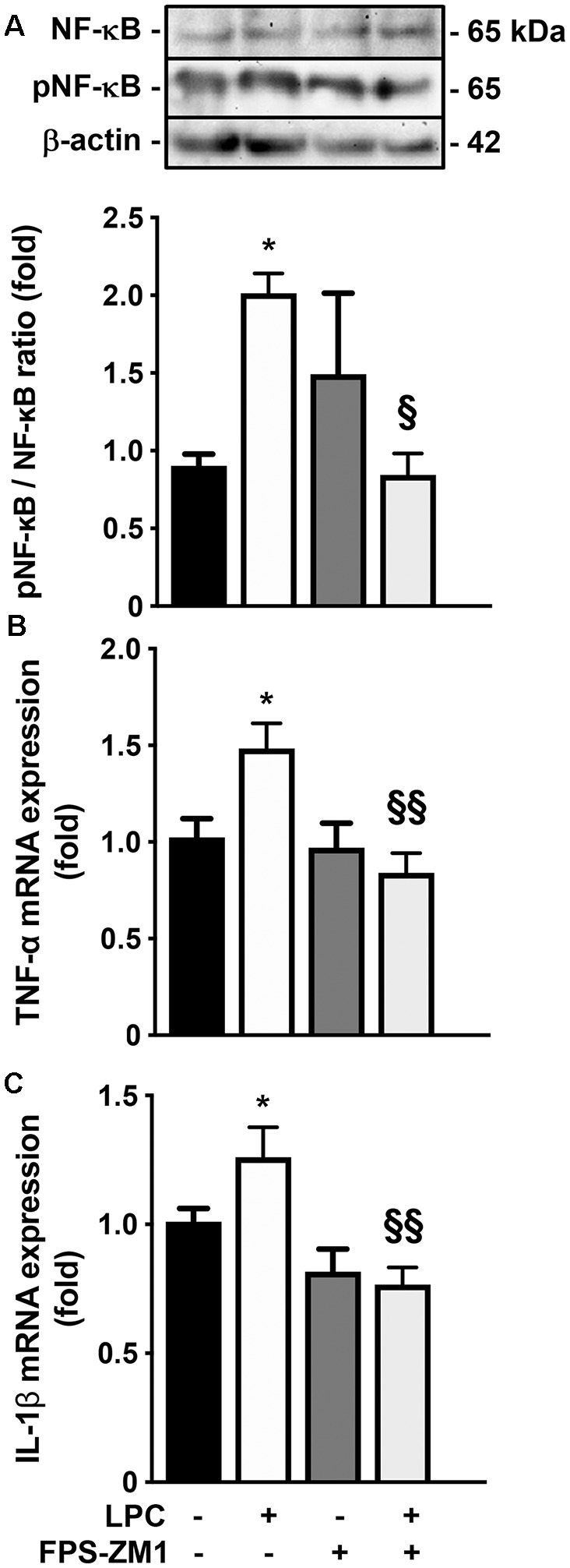
Inflammatory response induced by LPC is prevented by RAGE antagonist. OCSC were exposed to LPC (0.5 mg/ml) or LPC plus RAGE antagonist FPS-ZM1 (3 μM) at 7 days *in vitro* for 18 h. Following 30 h of recovery, **(A)** NF-κB activation was calculated by the ratio between protein expression of pNF-κB and NF-κB evaluated by Western blot. Gene expression of **(B)** tumor necrosis factor-α (TNF-α) and **(C)** interleukin-1β (IL-1β)was evaluated by qRealTime-PCR. Results are mean ± SEM from at least four independent experiments. One-way ANOVA with Bonferroni multiple comparison test was used for statistical significance (*p < 0.05 vs. Control; ^§§^*p* < 0.01 and ^§^*p* < 0.05 vs. LPC).

Given the observed gliosis, we next determined the inflammatory status, with particular focus on NF-κB, since it is one of the main executioners of the inflammatory response (Bianchi et al., 2010). NF-κB is held quiescent in the cytoplasm by binding to its inhibitory protein, IκB. Upon stimulation, IκB is rapidly phosphorylated, ubiquitinated, and then degraded by IκB kinases, resulting in the release and subsequent nuclear translocation of active NF-κB (O’Neill and Kaltschmidt, 1997). Importantly, NF-κB phosphorylation directs NF-κB transactivation and its action in the transcription of specific genes (Christian et al., 2016). Here, as depicted in Figure 3A; we observed that LPC-induced demyelination increased the activation of NF-κB, leading to a higher pNF-κB/NF-κB ratio (2.0-fold, *p* < 0.05), while co-exposure with RAGE antagonist FPS-ZM1 prevented that (0.9-fold, *p* < 0.05 vs. LPC). It is well established that the release of first-line pro-inflammatory cytokines, including TNF-α and IL-1β, occurs downstream NF-κB activation. As observed in Figures 3B,C, LPC stimulus increased both TNF-α and IL-1β mRNA expression (1.5- and 1.3-fold, *p* < 0.05, respectively), which was also abrogated by co-treatment with FPS-ZM1 (0.8-fold for both TNF-α and IL-1β, *p* < 0.01 vs. LPC).

Overall, our results indicate that the S100B–RAGE axis plays a crucial role in gliosis and inflammatory response following LPC-induced demyelination.

### RAGE Antagonist Treatment Prevents LPC-Induced Demyelination and Related Neuronal Impairment

**Figure 4:**
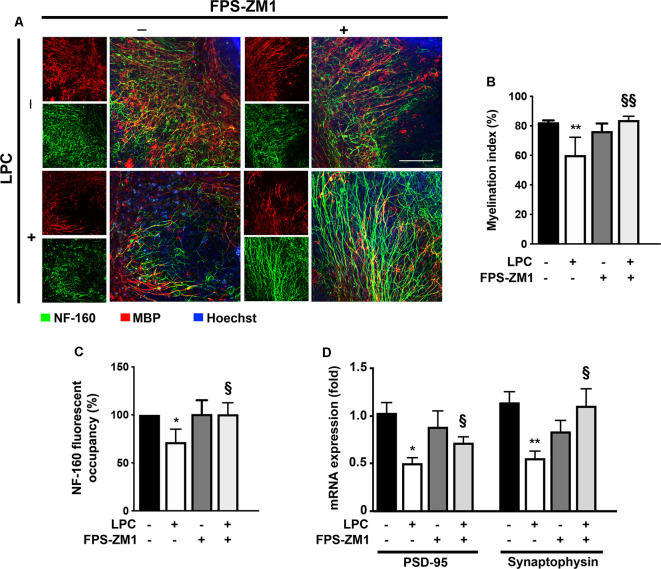
LPC-induced demyelination and impairment of neuronal integrity is prevented by RAGE antagonist. OCSC were exposed to LPC (0.5 mg/ml) or LPC plus RAGE antagonist FPS-ZM1 (3 μM) at 7 days *in vitro* for 18 h. Following 30 h of recovery, OCSC were immunostained for neurofilament-160 (NF-160) to detect neuronal axons (green) and myelin basic protein (MBP) to identify mature oligodendrocytes (OL, red) and stained with Hoechst to detect nuclei (blue). **(A)** Representative images are shown. Scale bar represents 100 μm. **(B)** The myelination index was calculated by the ratio between the area of colocalization of NF-160 and MBP and the total area occupied by NF-160. **(C)** Axonal integrity measured by NF-160 fluorescent occupancy. **(D)** Relative levels of synaptic markers (PSD-95 and synaptophysin) were determined by qRealTime-PCR. Results are mean ± SEM from at least four independent experiments. One-way ANOVA with Bonferroni multiple comparison test was used for statistical significance (**p < 0.01 and *p < 0.05 vs. Control; ^§§^*p* < 0.01 and ^§^*p* < 0.05 vs. LPC).

Several studies have already demonstrated that inflammation is closely associated with acute axonal injury and demyelination that further predisposes the axon to demise (Mandolesi et al., 2015). In addition, we have previously demonstrated in the same model of *ex vivo* demyelination that neutralization of S100B with a specific antibody partially prevents LPC-induced demyelination (Barateiro et al., 2016), highlighting the involvement of S100B in such condition. Here, we further evaluated whether S100B action in demyelination/remyelination was mediated by RAGE engagement. As shown in Figures 4A,B, LPC induced a marked decrease of the percentage of myelinated fibers (0.7-fold, *p* < 0.01) that was prevented by co-incubation with RAGE antagonist FPS-ZM1 (*p* < 0.01 vs. LPC), further confirming a crucial involvement of this S100B–RAGE axis in demyelination. Regarding demyelination-associated axonal damage, as depicted in Figures 4A–C, we observed that LPC-induced demyelination also decreased the area of NF-160 staining (0.7-fold, *p* < 0.05), indicating a reduced axonal integrity. However, when OCSC were treated with LPC in the presence of RAGE antagonist FPS-ZM1, neurofilament staining returned to Control values (*p* < 0.05 vs. LPC), suggesting axonal preservation due to direct prevention of axonal damage or as a consequence of decreased demyelination, since loss of the normal fine structure of myelin can cause axonal degeneration and even premature neuronal death. Next, we also evaluated gene expression of specific synaptic genes (Figure 4D) and observed that LPC-induced demyelination downregulated both postsynaptic density protein (PSD)-95 and synaptophysin (0.5-fold for both genes, *p* < 0.05). Interestingly, FPS-ZM1 treatment also reduced this inhibitory effect (0.7- and 1.0-fold, *p* < 0.05 and *p* < 0.01 vs. LPC, respectively).

Together, these results suggest that the inhibition of the S100B–RAGE axis may be protective against both demyelination and consequent axonal injury.

### LPC-Induced Impairment of Oligodendrocyte Differentiation Is Prevented by RAGE Antagonist

**Figure 5:**
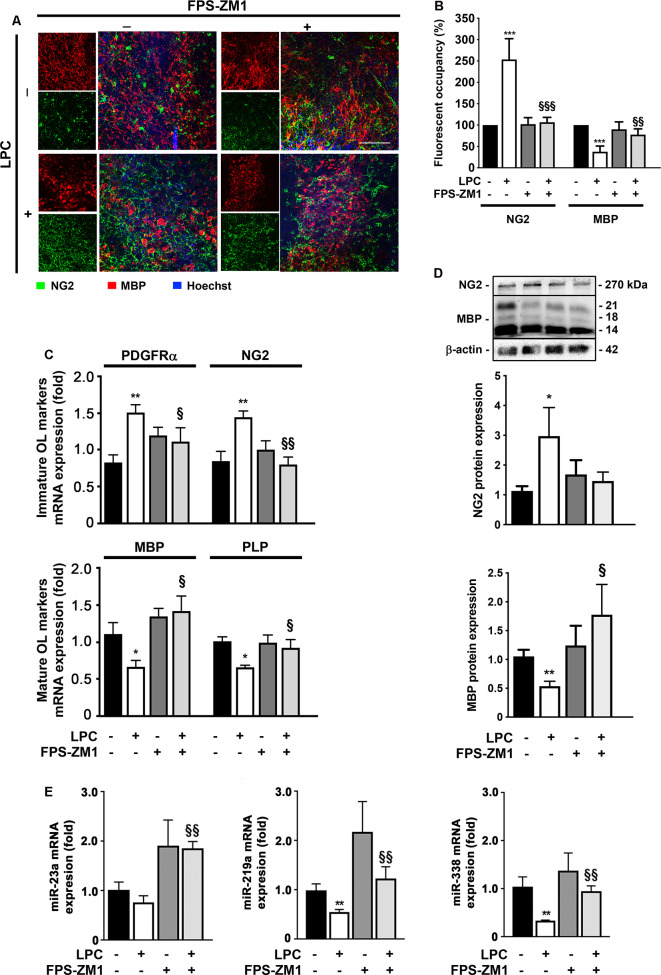
LPC-induced delay in oligodendrocyte (OL) maturation is prevented by RAGE antagonist. OCSC were exposed to LPC (0.5 mg/ml) or LPC plus RAGE antagonist FPS-ZM1 (3 μM) at 7 days *in vitro* for 18 h. Following 30 h of recovery, OCSC were immunostained to identify immature OL that express neural–glial antigen 2 (NG2, green) and mature OL that express MBP (red) and stained with Hoechst to detect nuclei (blue). **(A)** Representative images are shown. Scale bar represents 100 μm. **(B)** Quantification of the NG2 and MBP fluorescent occupancy.** (C)** Relative levels of the gene expression of the immature (PDFGRα/NG2) and mature (MBP/PLP) OL markers were determined by qRealTime-PCR. **(D)** Protein expression of NG2 and MBP was evaluated by Western Blot. Values are shown as mean ± SEM from at least four independent experiments performed. **(E)** Graph bars represent expression of miR-23a, miR-219a, and miR-338 evaluated by qRealTime-PCR following 30 h of recovery from LPC incubation. Results are mean ± SEM from at least four independent experiments. One-way ANOVA with Bonferroni multiple comparison test was used for statistical significance (***p < 0.001, **p < 0.01 and *p < 0.05 vs. Control; ^§§§^*p* < 0.01, ^§§^*p* < 0.01 and ^§^*p* < 0.05 vs. LPC).

Although considerable remyelination is achieved by endogenous OPC, during MS course, the extent and the quality of remyelination are limited. These limitations may arise from the failure of OPC to repopulate areas of demyelination and/or to differentiate into myelinating OL due to the presence of inhibitory factors and/or a lack of the stimuli required to induce the differentiation of these cells (Chang et al., 2002). Interestingly, in a previous report, we showed a toxic role of high S100B levels in OL development *via* RAGE activation, by reducing the transition from precursor cells to mature OL and the morphological maturation of differentiated OL (Santos et al., 2018). Therefore, we decided to explore in this *ex vivo* demyelinating model whether the S100B–RAGE axis could also be affecting OL differentiation/maturation that occurs following a demyelinating insult and is needed for proper remyelination. As indicated in Figures 5A,B, LPC-induced demyelination led to a significant decrease of fluorescent occupancy of mature OL expressing MBP (0.4-fold, *p* < 0.001), in parallel with an increase of immature ones stained for NG2 (2.5-fold, *p* < 0.001). Notwithstanding, co-treatment with RAGE antagonist FPS-ZM1 prevented these effects, increasing the MBP fluorescent occupancy (0.8-fold, *p* < 0.01 vs. LPC) and recovering NG2 fluorescent occupancy to Control values (*p* < 0.001 vs. LPC). In line with these results, we have also observed that LPC-induced demyelination increased the gene expression of immature OPC markers, such as platelet-derived growth factor receptor-α (PDGFR-α) and NG2 (1.6- and 1.5-fold, *p* < 0.01, respectively), while it decreased those of mature OL including MBP and myelin proteolipid protein (PLP; 0.7-fold for both, *p* < 0.05, Figure 5C). Co-incubation with RAGE antagonist FPS-ZM1 prevented LPC-induced effects by favoring the mRNA expression of mature markers (1.4- and 0.9-fold, *p* < 0.05 vs. LPC for MBP and PLP, respectively) and inhibiting the immature ones (1.1- and 0.8-fold, *p* < 0.05 and *p* < 0.01 vs. LPC, for PDGFRα and NG2, respectively). These results were corroborated by protein expression, as shown in Figure 5D. While LPC-induced demyelination increased NG2 protein expression (3-fold, *p* < 0.05) and decreased that of MBP (0.5-fold, *p* < 0.01), co-treatment with FPS-ZM1 prevented such effects for NG2 (1.5-fold) and increased MBP to values above Control (1.6-fold), which is significantly different from LPC treatment (*p* < 0.05).

Recent studies have demonstrated the importance of specific miRs, in regulating OPC differentiation and remyelination. In this context, miR-23a, miR-219a, and miR-338 were described as inducers of OL differentiation, being highly expressed in mature OL. The miRs are important not only for the transition of OPC to OL but also for the formation and maintenance of myelin (Dugas et al., 2010; Zhao et al., 2010; Lin et al., 2013). In this work (Figure 5E), miR-23a, miR-219a, and miR-338 were decreased in LPC-demyelinated OCSC (0.7-fold, non-significant; 0.5- and 0.3-fold, *p* < 0.01, respectively). Interestingly, LPC-induced demyelination in the presence of RAGE antagonist resulted in a complete abrogation of these miRs downregulation, improving their expression to levels similar to or even higher than those observed in Control experiments (1.8-, 1.1-, and 0.9-fold, *p* < 0.01, for miR-23a, miR-219a, and miR-338, respectively). These results suggest that prevention of RAGE engagement by released S100B may prevent LPC myelin damage and therefore maintain mature OL expression of these miRs and/or favor OL differentiation and maturation, and miRs expression, improving myelinogenesis and consequent remyelination.

Overall, these data indicate that inhibition of RAGE engagement may prevent LPC myelin destruction and/or promote OL differentiation and maturation, favoring a subsequent remyelination of axonal tracts.

### RAGE Antagonist Prevents LPC-Induced Demyelination and Favors Remyelination

**Figure 6:**
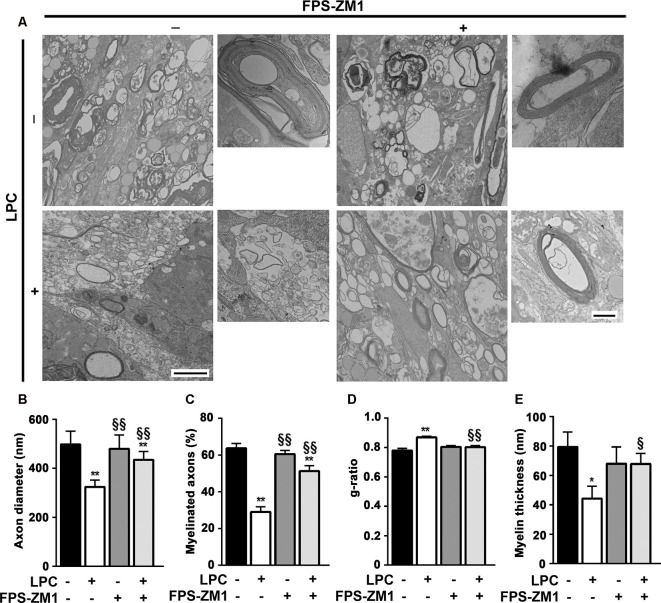
Inhibition of RAGE engagement prevents LPC-induced demyelination and favors remyelination. OCSC were exposed to LPC (0.5 mg/ml) or LPC plus RAGE antagonist FPS-ZM1 (3 μM) at 7 days *in vitro* for 18 h. Following 30 h of recovery, OCSC were fixed and observed by electron microscopy. **(A)** Representative electron micrographs of myelinated axons (scale bar equals 500 nm) and zoomed compact myelin layers (scale bar equals 100 nm) are shown. Images were analyzed to indicate **(B)** axon diameter for all axons, **(C)** percentage of myelinated axons per field, **(D)** *g*-ratio calculated by the ratio between axon diameter and fiber diameter, and **(E)** myelin thickness of all myelinated axons. Values are shown as mean ± SEM of at least three images from three different slices. One-way ANOVA with Bonferroni multiple comparison test was used for statistical significance (**p < 0.01 and *p < 0.05 vs. Control; ^§§^*p* < 0.01 and ^§^*p* < 0.05 vs. LPC).

**Figure 7:**
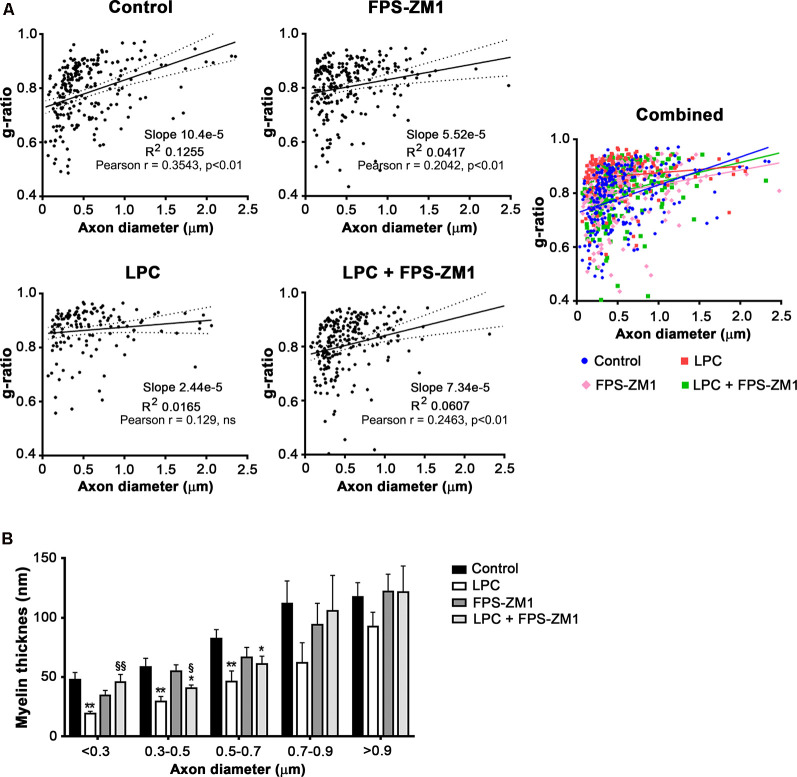
Inhibition of the S100B–RAGE axis favors remyelination. OCSC were exposed to LPC (0.5 mg/ml) or LPC plus RAGE antagonist FPS-ZM1 (3 μM) at 7 days *in vitro* for 18 h. Following 30 h of recovery, OCSC were fixed and observed by electron microscopy. **(A)** Scatter plot displays *g*-ratios of individual fibers as a function of their axon diameter. Pearson correlation coefficient was calculated for each condition. **(B)** Distribution of myelin thickness of individual fibers as a function of axon diameter. Values are shown as mean ± SEM of at least three images from three different slices. One-way ANOVA with Bonferroni multiple comparison test was used for statistical significance (**p < 0.01 and *p < 0.05 vs. Control; ^§§^*p* < 0.01 and ^§^*p* < 0.05 vs. LPC).

Spontaneous remyelination occurs in many MS lesions but, with time, may be inefficient, leading to shorter and thinner myelin sheaths. Moreover, since myelin sheaths have trophic functions for the axons, demyelination predisposes axons to secondary damage, while remyelination not only reduces neurodegeneration but also restores efficient electric conduction along axons (Nave, 2010). So, to better understand the role of the S100B–RAGE axis in myelin and axonal integrity, as well as remyelination upon LPC-induced demyelination, we performed electron microscopy. As shown in Figures 6A–C, LPC induced a decrease in the percentage of myelinated axons (0.5-fold, *p* < 0.01) and in axon diameter (0.7-fold, *p* < 0.01), which are possibly related to degenerating axons due to the lack of metabolic support of myelinating cells to neurons. In addition, as depicted in Figures 6A,D,E, the electron microscopy images revealed that sections from LPC-treated cultures have thinner myelin sheaths, when compared with Control ones (0.5-fold, *p* < 0.05), which is reflected in an increase in the *g*-ratio (1.1-fold, *p* < 0.01). However, OCSC demyelinated with LPC in the presence of RAGE antagonist FPS-ZM1 show a recovery in axon diameter (0.9-fold, *p* < 0.05 vs. LPC), in the percentage of myelinated axons (0.8-fold, *p* < 0.01 vs. LPC), and in myelin thickness (0.9-fold, *p* < 0.05 vs. LPC) and a decrease in the *g*-ratio to Control values (*p* < 0.01 vs. LPC). A further analysis of *g*-ratio plotted vs. axon caliber showed a significant correlation between them (*p* < 0.01) for all conditions except that of LPC-treated samples (Figure 7A), suggesting that myelination is dependent on axonal diameter also in OCSC and that demyelination impairs such correlation. More attractively, when we compared the linear regression of each condition (Figure 7A, combined), we observed that only LPC treatment elicited a significant different slope from Control samples (*p* < 0.01), corroborating the protective effect of FPS-ZM1 co-treatment. Interestingly, we also found that axons with lower axonal diameter (<0.7 μm) show significant thinner myelin upon LPC-induced demyelination (Figure 7B, *p* < 0.01), suggesting that those axons are the ones more prone to functional remyelination. Further, RAGE antagonism was able to significantly ameliorate myelin thickness of axons with lower caliber (<0.5 μm, *p* < 0.05), but still to values below Control samples, possibly indicating that this treatment besides preventing demyelination is also partially improving remyelination, giving rise to axons with a lower myelin thickness.

## Discussion

Excessive S100B concentrations have been identified in the CSF, serum, and *post-mortem* brain samples of MS patients (Barateiro et al., 2016). Accordingly, S100B is markedly released particularly during acute exacerbation, reaching maximal concentration and decreasing thereafter, being almost undetectable during the remission phase of the disease (Massaro et al., 1985; Missler et al., 1997). Curiously, S100B levels are significantly reduced in response to MS treatments such as mitoxantrone (Bartosik-Psujek et al., 2011) and nataluzimab (O’Connell et al., 2014), suggestive of an important role during MS pathogenesis. Moreover, its receptor RAGE is also found exacerbated in active lesions along the subcortical white matter or in corpus callosum (Barateiro et al., 2016), hippocampus (Sternberg et al., 2011), and spinal cord of MS patients (Yan et al., 2003). Using an *ex vivo* model of demyelination, we have also observed an increased expression of both S100B and its receptor RAGE following the demyelination stimulus of LPC, where S100B-enhanced expression was mainly determined by astrocytes (Barateiro et al., 2016). Curiously, as we have already observed in *post-mortem* samples of MS patients (Barateiro et al., 2016), also in the present study, RAGE expression shifted from cell nuclei to cytoplasm/cell membrane of microglia, suggesting an engagement of the S100B–RAGE axis. RAGE is expressed in many cell types, especially as an intracellular molecule that has to assemble to cytoplasmic membrane in order to allow ligand binding and subsequent activation of downstream signaling (Xie et al., 2008; Ramasamy et al., 2011). In fact, HMGB1, a RAGE ligand as S100B, is able to induce an increased expression of RAGE on the cell surface of human microvascular endothelial cells (Liaunardy-Jopeace et al., 2014) and umbilical vein endothelial cells (Doyle et al., 2012). Importantly, a very recent study demonstrated, in a mouse model of sterile musculoskeletal trauma, that inflammation induced by HMGB1 increases RAGE expression in both cytoplasm and plasma membrane, with cell surface expression being significantly increased in a time-dependent manner due to its activation (Zhong et al., 2020). Although RAGE engagement may occur due to the binding of HMGB1 (Sims et al., 2010), and indeed in a previous work, we have detected that OCSC from CD1 mice pups express high levels of HMGB1 following LPC-induced demyelination (Barateiro et al., 2016); in the present study, using OCSC from Wistar rat pups, no changes of HMGB1 gene expression were observed, indicative of a non-activated axis in the present demyelinating rat OCSC model. Moreover, it has been described that S100B extracellular action through RAGE engagement is able to promote its own expression (Donato et al., 2013). Interestingly, we observed that S100B is secreted after LPC incubation, even in the presence of the RAGE antagonist FPS-ZM1, but in this last condition, S100B–RAGE blockade is able to prevent later S100B or RAGE gene and protein expression. These results reinforce the role of FPS-ZM1 in preventing S100B acute and delayed effects in the neuroinflammatory milieu.

Along with demyelination, inflammation following exacerbated gliosis undoubtedly contributes to the pathogenesis of demyelinating lesions in MS. Our data showed that both astrogliosis and microgliosis accompanied the enhanced S100B–RAGE expression during the course of demyelination. Moreover, excessive extracellular S100B can induce an autocrine or paracrine glial activation *via* RAGE, leading to the transcription of pro-inflammatory genes (Donato et al., 2013), through the induction of NF-κB (Bianchi et al., 2010), intensifying the inflammatory response. It has been reported that S100B release by activated astrocytes is able to produce damage by causing overexpression of inducible nitric oxide synthase and subsequent release of nitric oxide (Bianchi et al., 2011), activating NF-κB in inflammatory response (Lam et al., 2001), or stimulating activation of microglia through a cytokine cycle (Bianchi et al., 2011). Accordingly, we observed a marked NF-κB activation and downstream TNF-α and IL-1β gene expression upon demyelination, which was prevented by co-exposure to RAGE antagonist FPS-ZM1. Thus, we may hypothesize that elevated concentration of S100B induced by demyelination may engage RAGE and promote astrocyte and microglia activation into an inflammatory phenotype, which in turn aggravates inflammation. These results are in line with a study on cerebral ischemia, showing that S100B induces microglia pro-inflammatory polarization with increased migration ability and inhibits anti-inflammatory polarization, in a process dependent of NF-κB activation (Zhou et al., 2018).

MS is increasingly recognized as a neurodegenerative disease, with primary or secondary neurodegeneration, as well as synaptopathy (Mandolesi et al., 2015). Over the course of the disease, demyelinated axons undergo irreversible degeneration, resulting in permanent disability, and correlate with disease progression (Franklin et al., 2012; Singh et al., 2013; Sorbara et al., 2014). Indeed, in *post-mortem* MS patient samples, the demyelinated hippocampi and cortex show decreased levels of proteins that are crucial to synaptic maintenance and function (Dutta et al., 2011). Additionally, the *in vivo* model of experimental autoimmune encephalomyelitis also exhibits structural synaptic alterations in several areas of the CNS, including the spinal cord, hippocampus, cerebellum, striatum, and cortex (Mandolesi et al., 2015). Regarding S100B, this small molecule represents a potential effector of inflammatory reactions and oxidative stress in neurons (Huttunen et al., 2000; Rothermundt et al., 2001). In our *ex vivo* demyelinating model, we observed not only the impairment of axonal tracts, but also the downregulation of specific pre- and post-synaptic marker gene expression. Both axonal loss and synaptic loss may be a consequence of the high S100B levels released in response to LPC treatment. In fact, a recent study demonstrated that intravitreal S100B injection had a direct destroying impact on the axons of the optic nerve, leading to neuronal apoptosis, decreased levels of βIII-tubulin, and numerous swollen axons (Kuehn et al., 2018). Moreover, deletion of S100B enhances hippocampal synaptic plasticity (Nishiyama et al., 2002), whereas excessive extracellular levels of S100B promote neuronal dysfunction or death in a direct manner (Mariggió et al., 1994), or as a result of gliosis-associated inflammatory response (Hu et al., 1997). Similarly, synaptic injury or loss has also been associated with astrogliosis (Zhu et al., 2003), which in our study was associated with enhanced S100B production. Together, this evidence suggests that the excessive S100B produced in response to demyelination may be implicated in neuronal and synaptic network dysfunction, either directly or through inflammatory damage, and prevented by the use of the specific RAGE antagonist FPS-ZM1.

Most attractively, in the present study, the use of RAGE antagonist prevented the loss of myelinated fibers induced by LPC, indicating that engagement of this receptor contributes to the extent of demyelination. These results are in agreement with our previous findings showing that neutralization of S100B has a beneficial role in the same model (Barateiro et al., 2016). Even though there is evidence that adult OL can contribute to myelin repair in the adult CNS (Duncan et al., 2018; Yeung et al., 2019), remyelination is thought to be mostly mediated by a pool of OPC that can differentiate into mature cells that then generate new myelin (Chang et al., 2002). Following activation, OPC undergo rapid proliferation in order to populate demyelinated areas (Woodruff et al., 2004). Besides, histopathological studies suggest that OPC are present in chronic MS lesions in the brain and spinal cord, and these cells seem to be relatively quiescent based on the lack of OL on the center of MS lesions (Chang et al., 2002).

S100B is expressed by OPC both in developing and adult mice brain (Deloulme et al., 2004) and in mature myelinating OL in brain and spinal cord (Hachem et al., 2005), being implicated in the regulation of their development. We recently showed that S100B–RAGE axis overactivation impaired neurodevelopmental *de novo* myelination (Santos et al., 2018), while S100B absence promoted a delayed OPC maturation following a demyelinating insult (Deloulme et al., 2004), suggesting that S100B effect may depend on the concentration locally attained. Accordingly, our results showed that LPC increased the number of precursor cells and decreased mature ones even after 30 h upon induced demyelination, indicating a delayed or impaired OPC differentiation into mature OL. These results are in line with previous reports indicating that, in MS patients, the remyelination is limited even though OPC are often efficiently recruited (Franklin, 2002). Interestingly, when we blocked the S100B–RAGE axis, we found a mature OL density similar to Control, suggesting a decrease in the mature OL damage or a promotion of remyelination.

Expression levels of miRs in OL lineage vary according to their differentiation stages. Moreover, differentiation of OPC, migration, and consequent remyelination are, among others, regulated by miRs that play an important role in regulating a large number of developmental and pathological processes (Qureshi and Mehler, 2012). Among these miRs, miR-23a, miR-219a, and miR-338 have been identified as critically involved in OL development, being preferentially and abundantly expressed in mature OL (Lau et al., 2008; Dugas et al., 2010; Zhao et al., 2010). Here, we observed a decrease in miR-23a, miR-219a, and miR-338 after demyelination induced by LPC, with these effects being counteracted by RAGE antagonist co-treatment. These results suggest that, in this model, increased number of mature OL and possibly remyelination, after inhibition of the S100B–RAGE axis, are at least in part due to changes in miRs expression. In fact, *in vitro* studies, showed that miR-23a and mir-219a levels are critical for OPC differentiation and enhance the proportion of mature OL (Lin and Fu, 2009; Dugas et al., 2010). Interestingly, *in vivo* increased levels of both miRs are able to induce OL differentiation and myelination/remyelination (Lin et al., 2013; Li et al., 2019). Of note, several studies have demonstrated that cooperation between miR-219a and miR-338 is necessary to promote OL maturation and myelination (Wang et al., 2017). Curiously, a recent study demonstrated that cooperation between miR-219a and miR-338 leads not only to enhanced OPC differentiation and maturation, but also to diminished microglial and astroglial activation with consequent decreased expression of pro-inflammatory cytokines (Nguyen et al., 2020).

Remyelination is an inefficient process in MS, which is likely due to an inhibitory microenvironment that prevents OPC from terminally differentiating into myelinating OL (Chang et al., 2002; Kuhlmann et al., 2008; Huang and Franklin, 2011). Indeed, remyelination remains an unmet therapeutic goal in MS in order to improve clinical deficits and to avert associated neurodegeneration. In fact, remyelination can restore conduction properties of axons, thereby restoring neurological function, and exerting a neuroprotective role on axons (Rodriguez, 2003; Sasaki et al., 2006; Waxman, 2006). Interestingly, our results suggest that blockade of the S100B–RAGE axis can partially improve remyelination, as observed by the presence of newly generated myelin sheaths thinner than normal, following LPC demyelination. This effect may be due to a direct effect on myelination or a result of reduced inflammatory milieu by co-treatment with the RAGE antagonist, improving OPC abilities to remyelinate. Nevertheless, the exact role of the S100B–RAGE axis should be further investigated.

Altogether, our results suggest that the high S100B levels released upon demyelination may engage the RAGE pathway contributing to MS pathogenesis, including impaired oligodendrogenesis and remyelination failure, neuronal loss and synaptic dysfunction, as well as glia activation with excessive neuroinflammatory microenvironment. Thus, blockade of S100B–RAGE interaction may be a potential new therapeutic strategy, namely for acute MS relapses where S100B is mostly elevated. This strategy may not only prevent the inflammatory-associated damage but also accelerate OL differentiation and remyelination, thus conferring protection of neuronal function in inflammatory lesions and collectively leading to a reduced CNS damage and a faster recovery.

## Data Availability Statement

The raw data supporting the conclusions of this article will be made available by the authors, without undue reservation.

## Author Contributions

AF conceived the project. AF, GS, and AB designed the experiments. GS and AB carried out the experiments, with AF’s contribution for organotypic cerebellar slice cultures. GS and AB analyzed the data and performed statistical analysis. GS, AB, DB, and AF wrote the manuscript. All authors contributed to the article and approved the submitted version.

## Conflict of Interest

The authors declare that the research was conducted in the absence of any commercial or financial relationships that could be construed as a potential conflict of interest.

## Supplementary Material

FIGURE S1 S100B is mainly expressed by astrocytes and receptor for advanced glycation end products (RAGE) by microglia, although RAGE expression shifts from a nuclear to a cytoplasmic expression upon lysophosphatidylcholine (LPC)-induced demyelination. Organotypic cerebellar slice cultures (OCSC) were exposed to LPC (0.5 mg/ml) or LPC plus RAGE antagonist FPS-ZM1 (3 μM) at 7 days *in vitro* for 18 h. Following 30 h of recovery OCSC were immunostained for S100B and astrocyte marker glial fibrillary acidic protein, GFAP, or for RAGE and microglia marker arginase-1 (Arg-1). Graph bars represent the percentage of area of colocalization of S100B in GFAP+ astrocytes **(A)** or RAGE in Arg1+ microglia **(B)**. **(C)** Representative images with higher magnification of RAGE (green), microglia (Arg-1, red) and Hoechst staining to detect nuclei (blue). Scale bar represents 30 μm. **(D)** Graph bars represent the ratio of RAGE fluorescence intensity between the nuclei and the cytoplasm. Results are mean ± SEM from at least three independent experiments. One-way ANOVA with Bonferroni multiple comparison test was used for statistical significance (**p* < 0.05 vs. Control).

FIGURE S2 High-mobility group box 1 (HMGB1) protein and gene expression is not altered by LPC-induced demyelination or RAGE antagonist treatment. Organotypic cerebellar slice cultures (OCSC) were exposed to LPC (0.5 mg/ml) or LPC plus RAGE antagonist FPS-ZM1 (3 μM) at 7 days *in vitro* for 18 h. Following 30 h of recovery, **(A)** protein expression of HMGB1 was evaluated by Western Blot and **(B)** relative levels of the gene expression by qRealTime-PCR. Results are mean ± SEM from at least four independent experiments. One-way ANOVA with Bonferroni multiple comparison test was used for statistical significance.
