# Supplementary figures and images for: S100B Impairs Oligodendrogenesis and Myelin Repair Following Demyelination Through RAGE Engagement
Source: Front Cell Neurosci. 2020 Sep 4;14:279. doi: 10.3389/fncel.2020.00279 (PMC7500156; doi:10.3389/fncel.2020.00279)

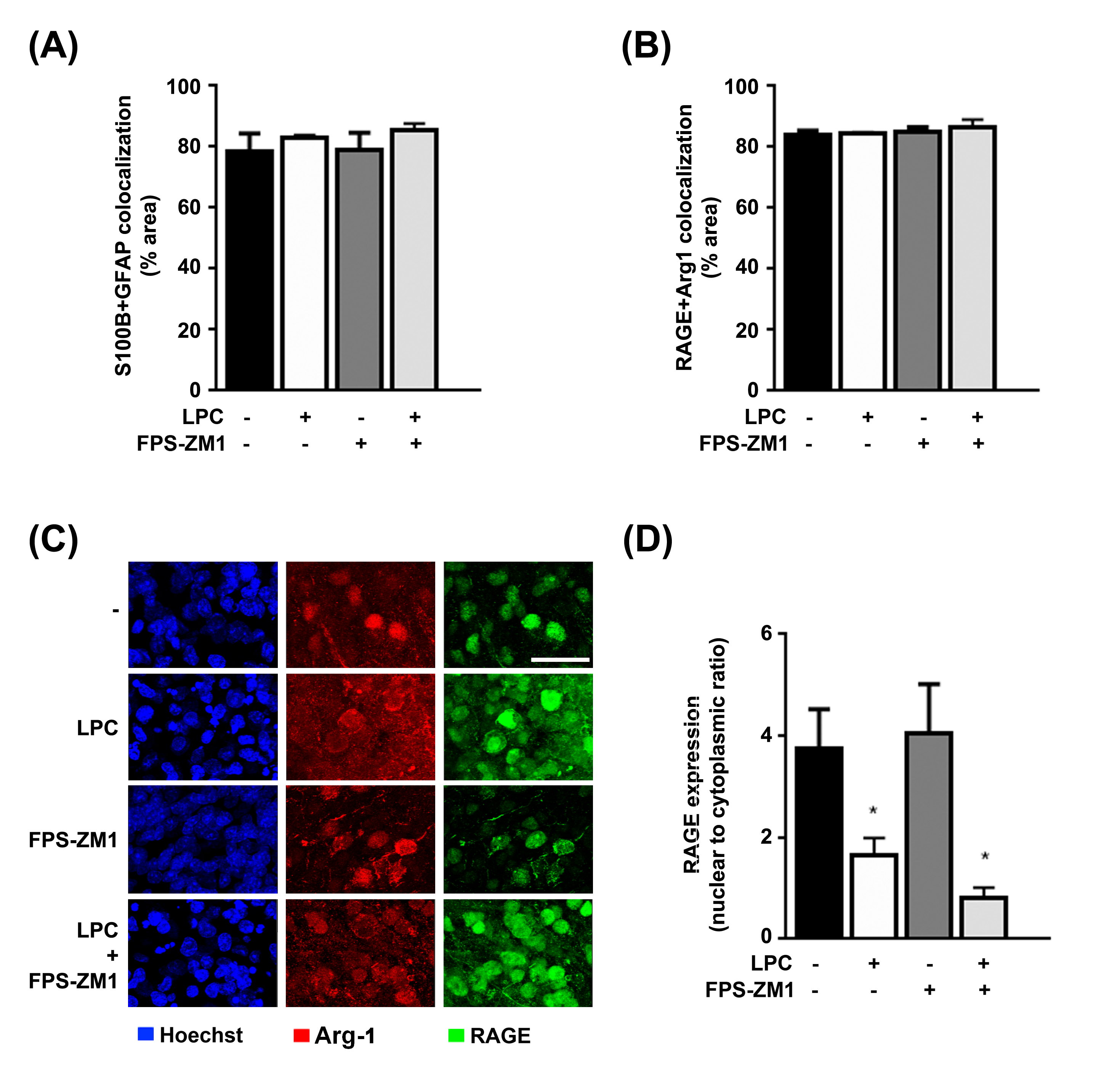

Supplement: FIGURE S1 — S100B is mainly expressed by astrocytes and receptor for advanced glycation end products (RAGE) by microglia, although RAGE expression shifts from a nuclear to a cytoplasmic expression upon lysophosphatidylcholine (LPC)-induced demyelination. Organotypic cerebellar slice cultures (OCSC) were exposed to LPC (0.5 mg/ml) or LPC plus RAGE antagonist FPS-ZM1 (3 μM) at 7 days in vitro for 18 h. Following 30 h of recovery OCSC were immunostained for S100B and astrocyte marker glial fibrillary acidic protein, GFAP, or for RAGE and microglia marker arginase-1 (Arg-1). Graph bars represent the percentage of area of colocalization of S100B in GFAP+ astrocytes (A) or RAGE in Arg1+ microglia (B). (C) Representative images with higher magnification of RAGE (green), microglia (Arg-1, red) and Hoechst staining to detect nuclei (blue). Scale bar represents 30 μm. (D) Graph bars represent the ratio of RAGE fluorescence intensity between the nuclei and the cytoplasm. Results are mean ± SEM from at least three independent experiments. One-way ANOVA with Bonferroni multiple comparison test was used for statistical significance (*p < 0.05 vs. Control). [file Image_1.TIF]

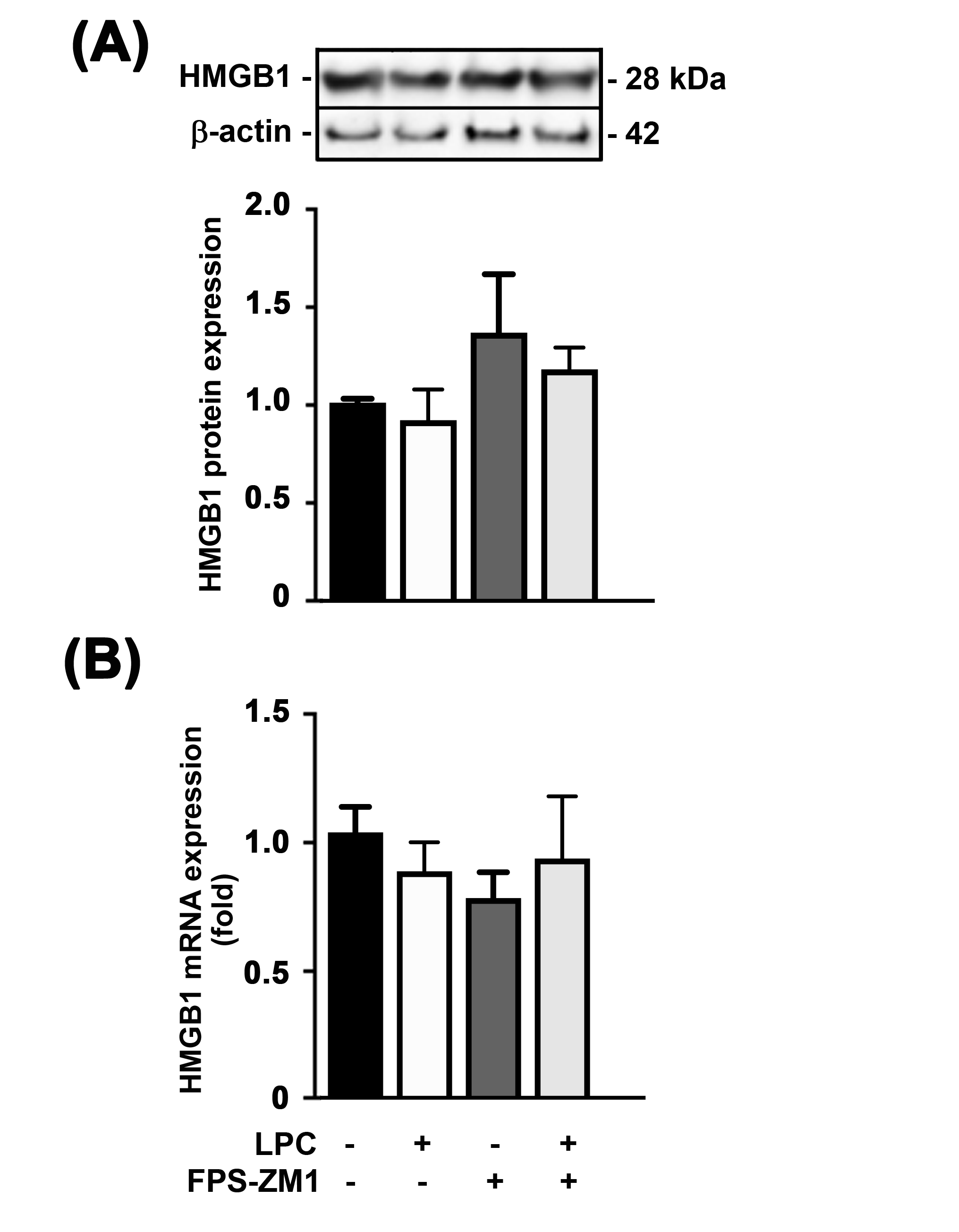

Supplement: FIGURE S2 — High-mobility group box 1 (HMGB1) protein and gene expression is not altered by LPC-induced demyelination or RAGE antagonist treatment. Organotypic cerebellar slice cultures (OCSC) were exposed to LPC (0.5 mg/ml) or LPC plus RAGE antagonist FPS-ZM1 (3 μM) at 7 days in vitro for 18 h. Following 30 h of recovery, (A) protein expression of HMGB1 was evaluated by Western Blot and (B) relative levels of the gene expression by qRealTime-PCR. Results are mean ± SEM from at least four independent experiments. One-way ANOVA with Bonferroni multiple comparison test was used for statistical significance. [file Image_2.TIF]
